# Validation of the Turkish version of the assessment of systemic sclerosis-associated Raynaud’s phenomenon (ASRAP)

**DOI:** 10.55730/1300-0144.6088

**Published:** 2025-08-19

**Authors:** Duygu TEMİZ KARADAĞ, Şeyma YILMAZ, Betül DİKKANOĞLU DEMİROK, John D. PAULİNG, Ayten YAZICI, Ayse ÇEFLE

**Affiliations:** 1 Division of Rheumatology, Department of Internal Medicine, Faculty of Medicine, Kocaeli University, Kocaeli, Turkiye; 2 Department of Internal Medicine, Faculty of Medicine, Kocaeli University, Kocaeli, Turkiye; 3 Department of Rheumatology, North Bristol NHS Trust and Bristol Medical School, University of Bristol, Bristol, UK

Dear Editor,

Raynaud’s phenomenon (RP) contributes significantly to patient morbidity and is closely associated with digital ulcers, pain, and functional impairment [1–3]. Accurate assessment of RP severity is critical for evaluating treatment response and improving patient quality of life. Evaluating RP presents challenges due to the episodic nature of the symptoms. Traditional patient-reported outcome (PRO) tools, such as the Raynaud’s condition score (RCS), often fail to fully capture the physical, emotional, and functional impact of RP on patients [4,5]. The assessment of systemic sclerosis-associated Raynaud’s phenomenon (ASRAP) questionnaire was recently developed to address these limitations by prioritizing patient experience [6,7].

We aimed to translate and validate the Turkish version of the ASRAP Short Form (ASRAP-SF), a 10-item tool intended for both clinical and research use. The Turkish version of the ASRAP-SF was developed following established cross-cultural adaptation guidelines. A total of 86 patients with systemic sclerosis (SSc) fulfilling the 2013 ACR/EULAR criteria [8] completed the ASRAP-SF, the scleroderma health assessment questionnaire (SHAQ) [9], and the SF-36v2 [10]. A total of 47 patients completed the ASRAP-SF again after a mean interval of 48.4 days for test–retest reliability analysis.

The Turkish ASRAP-SF demonstrated excellent internal consistency (Cronbach’s alpha = 0.946, 95% CI: 0.927–0.962) and good test–retest reliability (ICC = 0.749, 95% CI: 0.525–0.867). Similar to the original validation study, patient participation was restricted to the winter months [7]. The longer test–retest interval in our study, compared to the original (1–2 months versus 1 week), did not compromise reproducibility, likely due to the stable disease activity of participants.

Convergent validity was confirmed through moderate to strong correlations with HAQ-DI (r = 0.594), SHAQ RP-VAS (r = 0.675), SHAQ DU-VAS (r = 0.399), and SHAQ overall severity visual analog scale (VAS) (r = 0.468), consistent with previous reports (Figure). In our study, we observed a weak but significant correlation with the SF-36, which is frequently used in research to broadly assess general daily activities, pain, social relationships, daily functions, and emotional well-being. The weak correlations between the ASRAP-SF and SF-36 domains suggest the instrument’s specificity for RP rather than overall health status. This result suggests that the ideal scale for assessing RP should be capable of comprehensively evaluating its physical, emotional, and functional impact on patients.

ASRAP-SF scores were significantly higher among patients with vasculopathy-related complications, including active digital ulcers (54.9 ± 11.6 versus 47.1 ± 9.98, p = 0.011), digital ulcer history (51.2 ± 9.94 versus 46.3 ± 10.86, p = 0.037), digital amputations (56.8 ± 8.7 versus 47.7 ± 10.5, p = 0.023), and a scleroderma pattern on nailfold capillaroscopy (50.2 ± 10.3 versus 43.7 ± 8.94, p = 0.005) (Table). These findings support the discriminant validity of the ASRAP-SF and align with known microvascular complications in SSc. This result is consistent with the RCS, where scores were higher in patients with digital ulcers compared to those without [4].

To our knowledge, this is the first validation of ASRAP-SF in a language other than English. This study indicates that the questionnaire suits SSc patients from diverse geographic, cultural, and ethnic backgrounds, representing the first validation of ASRAP-SF in a language other than English. Our findings suggest that the Turkish version is a valid and reliable PRO tool for assessing the severity and impact of SSc-RP and may be used in clinical trials and longitudinal cohort studies.

## Figures and Tables

**Figure:**
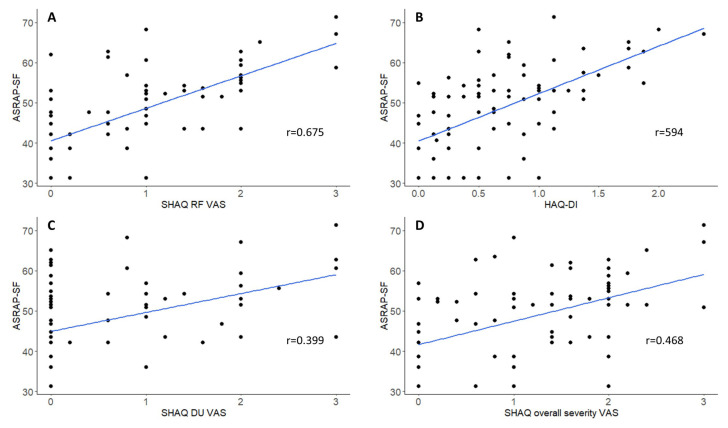
Correlation between ASRAP and legacy instruments for assessing SSc-RP. Scatterplots are annotated with lines of best fit and Pearson’s rho values, demonstrating the correlation between ASRAP (y-axis on each plot) and (A) SHAQ RP-VAS, (B) HAQ-DI, (C) SHAQ DU-VAS, and (D) SHAQ overall severity VAS. SHAQ, scleroderma health assessment questionnaire; VAS, visual analog scale; RF-VAS, Raynaud’s phenomenon visual analog scale; DU-VAS, digital ulcer visual analog scale.

**Table:** Impact of clinical phenotype, autoantibodies, and nailfold capillary abnormalities on ASRAP-SF scores.

		ASRAP-SF	p (T-test)

Active DU	Yes	54.9 ± 11.6	0.011
	No	47.1 ± 9.98	
DU*	Yes	51.2 ± 9.94	0.037
	No	46.3 ± 10.86	
Digital amputation	Yes	56.8 ± 8.7	0.023
	No	47.7 ± 10.5	
PHT	Yes	52.3 ± 11	0.471
	No	48.3 ± 10.7	
ILD	Yes	47.6 ± 10.2	0.347
	No	49.3 ± 11.1	
Disease subtype	diffuse	50.3 ± 11.3	0.308
	limited	47.9 ± 10.9	
Autoantibody	Anti-Scl70 positive	48.6 ± 10.1	0.757
	Anti-Scl70 negative	48.4 ± 10.9	

Capillaroscopy	SD pattern	50.2 ± 10.3	0.005
	Non-SD pattern	43.7±8.94	

ILD, interstitial lung disease; PHT, pulmonary hypertension; DU, digital ulcer; SD, scleroderma; ASRAP-SF, assessment of systemic sclerosis-associated Raynaud’s phenomenon – Short Form.

* Patients who had digital ulcers at any time.
